# Human fetal mesenchymal stem cells secretome promotes scarless diabetic wound healing through heat‐shock protein family

**DOI:** 10.1002/btm2.10354

**Published:** 2022-06-21

**Authors:** Bin Wang, Mengru Pang, Yancheng Song, Haixing Wang, Pan Qi, Shanshan Bai, Xiaoxuan Lei, Shikun Wei, Zhixian Zong, Sien Lin, Xiaoting Zhang, Xiaotong Cen, Xia Wang, Yongkang Yang, Yuan Li, Yan Wang, Hongjie Xu, Lin Huang, Micky Tortorella, Biao Cheng, Yukwai Lee, Dajiang Qin, Gang Li

**Affiliations:** ^1^ Innovation Centre for Advanced Interdisciplinary Medicine, Key Laboratory of Biological Targeting Diagnosis, Therapy and Rehabilitation of Guangdong Higher Education Institutes The Fifth Affiliated Hospital of Guangzhou Medical University Guangzhou China; ^2^ The Chinese University of Hong Kong (CUHK)‐Guangzhou Regenerative Medicine and Health Guangdong Laboratory (GDL) Advanced Institute for Regenerative Medicine Bioland Laboratory (Guangzhou Regenerative Medicine and Health Guangdong Laboratory) Guangzhou China; ^3^ Department of Burn and Plastic Surgery The Affiliated Hospital of Guizhou Medical University Guiyang Guizhou China; ^4^ Department of orthopedics The Affiliated Hospital of Guangdong Pharmaceutical University Guangzhou China; ^5^ Department of Orthopaedics and Traumatology, Stem Cells, and Regenerative Medicine Laboratory Li Ka Shing Institute of Health Sciences, The Chinese University of Hong Kong, Prince of Wales Hospital Shatin Hong Kong; ^6^ Department of Oral and Maxillofacial Surgery/Pathology Amsterdam UMC and Academic Center for Dentistry Amsterdam (ACTA), Vrije Universiteit Amsterdam, Amsterdam Movement Science Amsterdam The Netherlands; ^7^ Department of Plastic Surgery General Hospital of Southern Theater Command, PLA Guangzhou China; ^8^ Division of Plastic, Reconstructive and Aesthetic Surgery, Department of Surgery The Chinese University of Hong Kong, Prince of Wales Hospital Shatin Hong Kong; ^9^ Centre for Regenerative Medicine and Health Hong Kong Institute of Science and Innovation, Chinese Academy of Sciences Hong Kong China

**Keywords:** bioreactor, diabetic wound healing, fetal mesenchymal stem cell secretome, PLGA particles, quality control

## Abstract

The high mortality rate of patients with diabetic foot ulcers is urging the appearance of an effective biomedical drug. Senescence is one of the major reasons of aging‐induced decline in the diabetic wound. Our previous studies have demonstrated the anti‐senescence effect of secretomes derived from human fetal mesenchymal stem cells (hfMSC). The present study tends to explore the potential role of hfMSC secretome (HFS) in wound healing through anti‐aging. Meanwhile, we try to overcome several obstacles in the clinical application of stem cell secretome. A verticle bioreactor and microcarriers are employed to expand hfMSC and produce the HFS on a large scale. The HFS was then subjected to lyophilization (L‐HFS). The PLGA (poly lactic‐*co*‐glycolic acid) particles were used to encapsulate and protect L‐HFS from degradation in the streptozotocin (STZ)‐induced diabetic rat model. Results showed that HFS‐PLGA significantly enhanced wound healing by promoting vascularization and inhibiting inflammation in the skin wound bed. We further analyzed the contents of HFS. Isobaric tag for relative and absolute quantitation (ITRAQ) and label‐free methods were used to identify peptides in the secretome. Bioinformatics analysis indicated that exosome production‐related singling pathways and heat‐shock protein family could be used as bio‐functional markers and quality control for stem cell secretome production.

## 1 INTRODUCTION

Diabetes mellitus is one the most common metabolic diseases leading to impaired chronic wound conditions. The 5‐year mortality rate for patients suffering from DFU (diabetic foot ulcer) is even higher than that of prostate or breast cancer.^1^, ^2^, ^3^ Despite the great clinical demand, there is only one biological drug (recombinant human PDGF‐BB, Regranex Ltd. Co, USA) and two biologic/devices (Apligraf™ and Dermagraft™) have been approved by the US Food and Drug Administration over the past 15 years^4^ for DFU treatment. DFU is an aging‐related disease. Senescence, also known as cellular aging has drawn a lot of attention recently. Senescent cells usually lose their functions and induce inflammation of the vicinity normal cells by SASP (senescence associate secretory pattern).^5^ Eliminating or prohibiting senescence is a promising strategy to promote wound healing. The function of stem cells is mainly attributed to the cells and their secreted factors. Physical interaction is important because mesenchymal stem cells (MSC) regulates macrophages/fibroblast proliferation and differentiation in tissue repair through physical interaction.^6^ Besides, various studies have proved that stem cells could exert their function through paracrine signals. Our study makes use of the secreted factors of the stem cells to mimic the regenerative microenvironment in the wound area. The secretome inherited the pro‐angiogenesis and immune‐modulation ability of the stem cells in our study. Recently, it is recognized that the various positive effects of MSCs are mainly attributed to their paracrine factors in vivo.^7^ The function of the secretome is differed and depends on the origin of the cell.^8^ Human first trimester (within 3‐month implantation) mesenchymal stem cells (hfMSC) exhibited longer telomere and higher activity of telomerase allowing prolonged culture in vitro.^9^ Our previous study found that transferring only one mitochondrion from hfMSC to the recipient cell is sufficient to induce anti‐aging gene expression.^10^ The secretome derived from hfMSC showed an anti‐senescence effect on adult cells.^11^, ^12^ Meanwhile, in contrast to salamanders who regenerate whole limbs after amputation even in adulthood, scarless healing in humans is only seen in fetal skins.^13^, ^14^ The fetal wound healing process is different from the adult in many aspects, such as growth factors, gene expression profiles, extracellular matrix (ECM), and inflammatory responses.^15^, ^16^, ^17^ Thus, studying the unique proteins or factors of hfMSC secretome (HFS) may shed a light on the scarless healing in diabetic wounds.

Several studies have proven the effect of cell secretomes on tissue repair and regeneration.^18^, ^19^, ^20^, ^21^, ^22^ Stem cell secretomes hold great potential in clinical application. The present study focus on various obstacles that stand in the way from bench to bedside. The traditional monolayer culture system is not sufficient to meet the requirement of the amount of the secretome on a large scale. The limiting surface area in monolayer culture reduces the production rate of secretome as well as prolongs the time to expand the stem cell. The bioreactor is an ideal choice for scaling up the manufacturing of HFS. The presence of microcarriers provides enough surface areas for cell attachment while shear stresses caused by agitation stimulate cell proliferation.^23^ Phosphate buffered saline (PBS) Biotech (California, USA) has developed a vertical‐peddle bioreactor providing optimized stir power and liquid shear force to the cells on microcarriers,^24^ which has been used for the successful expansion of MSCs and inducible pluripotent stem cells.^25^, ^26^ The PBS bioreactor system was used in the present study, to produce HFS on a large scale. MSCs released at least 44 biological factors (include TIMP2, FAS, MIP‐3b, TRAIL R4, etc).^27^ The bioactivities of the factors are of most importance in contributing to the function of the secretome. Reciprocal freeze and thaw of secretome during storage and transportation may compromise the function of HFS. Freeze‐drying is the most wide‐accepted method to preserve the activity of protein drugs.^28^ In our study, the bioreactor‐produced HFS is subject to lyophilization making it become an off‐the‐shelf product.

Systematic injection of secretome will require a large amount of the factors while the side effect is hard to predict. Thus, local administration of secretome as a free drug is the most efficient way to achieve effective dosage. However, the retention time of drugs in the wound area is limited due to the blood flow and enzyme degradation. Biomaterial‐based particles have been used to retain and release the drug over time. Polymeric particle is the optimized option that can reach the therapeutic effect with reduced the effective dosage.^29^ PLGA (poly lactic‐co‐glycolic acid) particle has been used for the controlled delivery of small molecule drugs and proteins. Especially, PLGA encapsulated drugs have been widely used in clinics for reducing inflammation.^30^ In our study, we encapsulated HFS in PLGA which is a mature drug release system to retain the bioactivity of HFS and prolonged the required amount of secretome during wound healing.

Recently, a study summarized clinical studies related to secretomes which can be found on clinical.gov, only three of them were completed without the availability of the result. Until now, there is no clear regulations have been stated by FDA. The age of the donor or the tissue sources of the cell is different, leading to the composition of the secretome depending on the variable cell source.^31^ Allogenic secretome therapy requires the standardization of internal quality control to calibrate its effective dosage as the inter‐donor variation cannot guarantee the therapeutic effect. In the present study, we compare the proteomic profile of the secretome from fetal stem cells and adult stem cells for anti‐aging‐related proteins which can explain the superior function of the HFS. We used a high throughput proteomic method (ITRAQ) to compare HFS with HAS (human adult stem cell secretome). The data were analyzed using Cytoscape and ClueGO for bioinformatics prediction,^32^ Our study identified that the heat‐shock protein family showed their pivotal role in promoting wound healing and angiogenesis. These factors have the potential to be biomarkers and quality control of secretome production on large scale.

## 2 RESULTS

### 2.1 Large‐scale expansion of hfMSC in vertical‐wheel bioreactor

**FIGURE 1:**
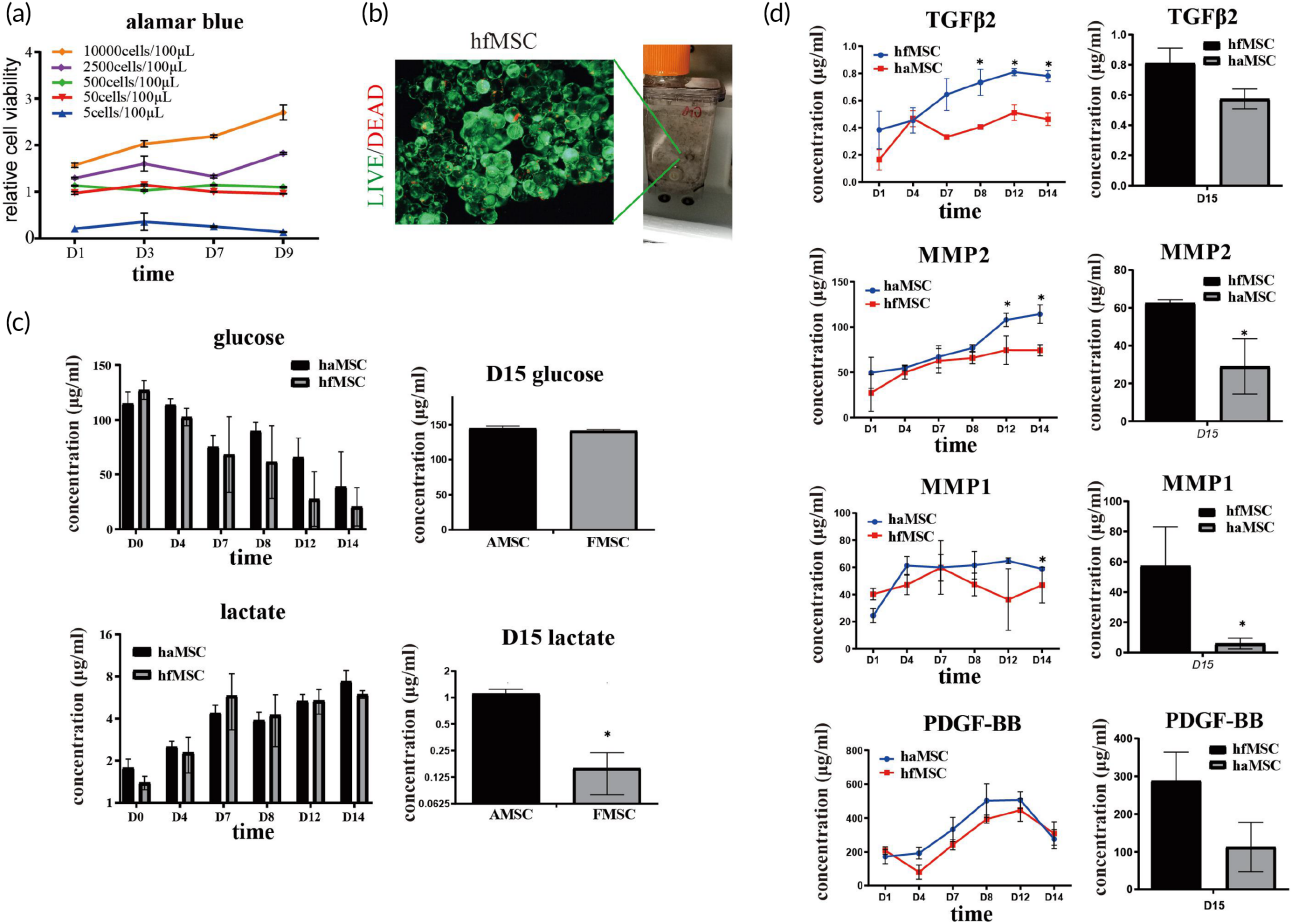
Large scale expansion of human fetal mesenchymal stem cells (hfMSCs) in a vertical‐wheel bioreactor. (a) Cell viability of various density of cells was measured using Alamar blue test. The data were normalized to negative control of all time points. Starting cell density was 1 × 106 cells/100 ml. *N* = 3; (b). Live/dead cell staining results showed that fetal MSCs and microcarriers formed aggregates and more than 90% of the cells were alive at Day 14 culture; (c). Cell metabolic index changes during culture. The glucose concentration kept on decreasing during culture, indicating that cells were actively proliferating. After change into serum‐free media, at Day 15, lactate concentration was lower in the hfMSCs culture suggesting hfMSC have a unique pattern of energy metabolism, *N* = 3; (d). During 14 days culture, ELISA results showed that the concentration of TGFβ2, VEGF, MMP1, and MMP2 were significantly higher in media of hfMSCs than those in haMSCs media; Statistic: Mann–Whitney *U* (unpaired, nonparametric *T*‐tests). *N* = 3, *p < 0.5. Brightness and contrast were adjusted for clear demonstrated contents of the representative pictures. TGFβ2, Transforming growth factor beta‐2; VEGF, Vascular endothelial growth factor; MMP, matrix metalloproteinase.

We use a vertical‐wheel bioreactor combined with collagen‐coated microcarriers. We first optimized the ratio of the number of hfMSC to the weight of microcarriers. A series of cell densities were tested. The various numbers of hfMSCs and the microcarrier were mixed and co‐cultured in 96 well plates. The Alamar blue result indicated that when cell density was higher than 500 cells/100 μl medium (equal to 500 cells/0.03 g microcarriers), the hfMSCs kept proliferating at least for 1 week (Figure 1a). We, therefore, chose 1000 cell/0.03 g microcarriers as our standard culture condition. And the culture medium will be half changed every 7 days. Usually, 1–2 weeks of culture is sufficient for hfMSCs and microcarriers to form visible aggregates, live/dead cell staining showed that more than 90% of cells were alive (Figure 1b). In bioreactor culture, the formation of the aggregates suggested that the culture system has reached its limits. A beads‐to‐beads passage is required to expand the culture scale. In 100 ml reaction volume, about 5 × 10^6^ human bone marrow mesenchymal stem cells were seeded. The cell number increased to nearly 3 × 10^7^ cells after 10‐day cultures. We then calculate the doubling time of hfMSC based on the number of cells. The result shows that, in bioreactor culture, the doubling time of hfMSC is around 36 h which is similar to that of the monolayer culture.

Thus, we choose 2 weeks as our endpoint to collect the secretome when we observed the formation of the aggregates. To monitor the metabolic status of cells during culture, we tested the glucose and lactate concentration of the culture media. Human adult mesenchymal stem cells (haMSCs) were used as control. Compared to haMSCs, hfMSCs utilized more glucose during culture, indicating that hfMSCs have a more active metabolic status. On Day 15 in culture, despite the cell numbers of hfMSCs being significantly higher than that of haMSCs, they only produced 1/10 of lactate in contrast to that of haMSC (Figure 1c). We performed an ELISA test to measure several well‐established factors during 14 days of culture. hfMSCs produced more TGF‐β2, PDGF‐BB, MMP1, and MMP2 in contrast to haMSCs at all‐time points (Figure 1d). Therefore, hfMSCs have higher proliferation ability and higher metabolic rate, they could produce higher concentrations of growth factors in HFS. The collected secretome was then subjected to lyophilization to keep the bioactivity of the factors.

### 2.2 Lyophilized HFS treatment rejuvenated human adult skin cells into a fetal skin cells‐like phenotype

**FIGURE 2:**
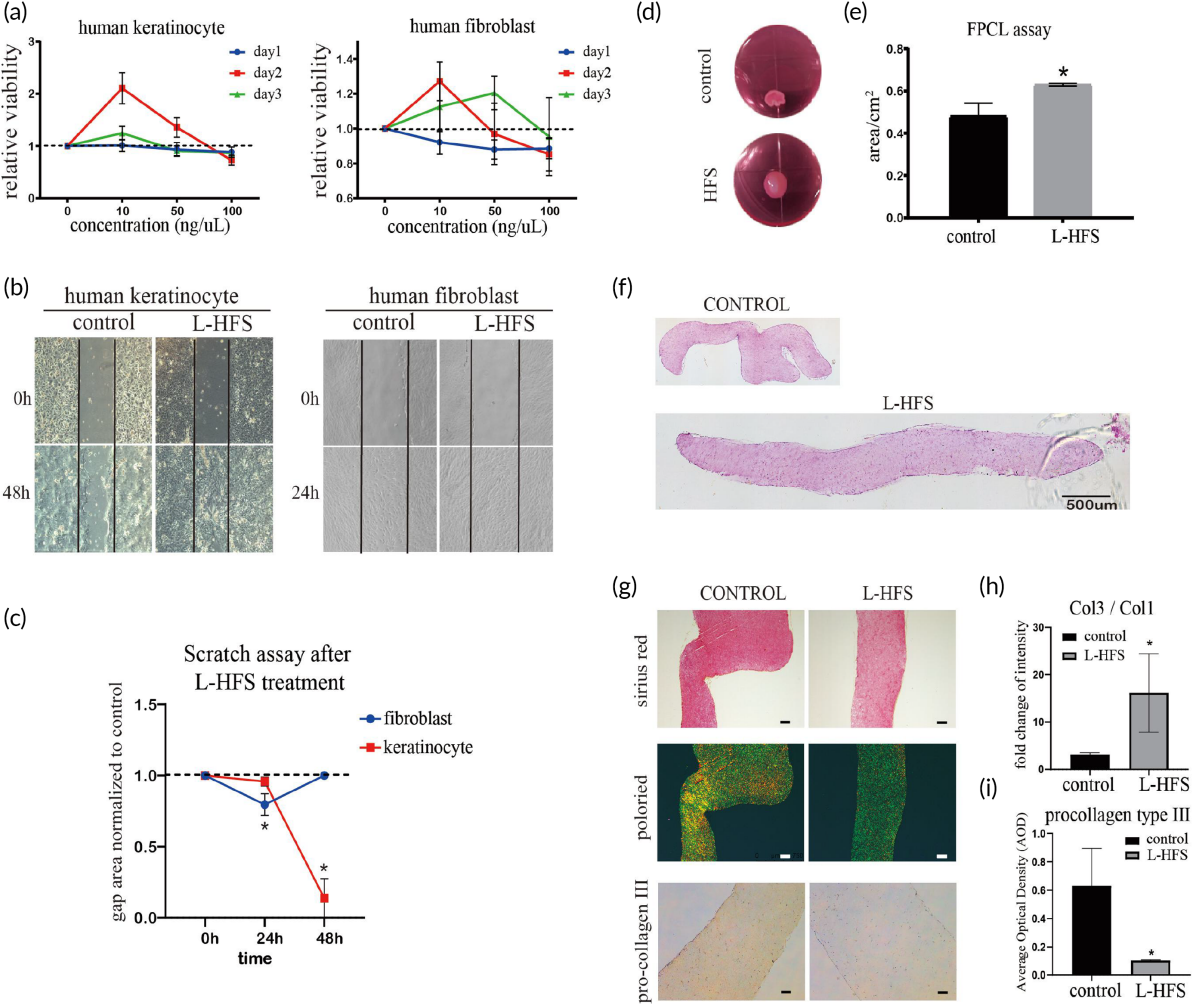
Lyophilized human fetal mesenchymal stem cell secretome (L‐HFS) rejuvenated human adult skin cells into a fetal skin cells‐like phenotype. (a) Effects of L‐HFS on the proliferation of human skin cells. L‐HFS promoted cell proliferation of human keratinocytes and slightly inhibited the cell proliferation of human fibroblasts at Day 1, but the cell proliferation of fibroblasts was enhanced at Day 3; (b) Scratch assay results demonstrated that L‐HFS significantly promoted gaps closure of human keratinocytes and fibroblasts. (c) Quantitative result of the gap area. The data represent two‐time points, 24 and 48 h after scratch. Areas were calculated by image J. *N* = 3, *p < 0.05; (d) Fibroblast populated collagen lattice (FPCL) assay showed that HFS treatment reduced the shrinkage of FPCL. The surface area was measured by image J. (e) Quantitative results of FPCL surface area, *n* = 3, *p < 0.05; (f) Gross view of H&E staining of the FPCL slice. The morphology of L‐HFS group is flattened while the control group appear obvious contraction. Scale bar = 500 μm; (g) Sirius red staining and polarized microscopy examination showed that there was more green color in the L‐HFS treatment group compared with the control group, indicating the higher intensity of type III collagen. The green color indicates type III collagen, while the yellow color indicates type I collagen; (h and i) Ratio of type I collagen to type III collagen based on the color intensity was measured by image J. Immunohistochemistry staining showed decreased expression of pro‐type III procollagen suggesting the maturation of type III collagen was enhanced after L‐HFS treatment. Scale bar = 200 μm; Statistic: Mann–Whitney *U* (unpaired, nonparametric *T*‐tests). *N* = 3, *p < 0.5. Brightness and contrast were adjusted for clear demonstrated contents of the representative pictures

The migration and proliferation of skin cells are crucial for skin regeneration. The effects of lyophilized HFS (L‐HFS) on human keratinocytes and fibroblasts migration and proliferation were tested. Alamar blue assay was used to test the optimal dosage of L‐HFS. An effective concentration window (10–50 ng/μl) was selected as the optimal dosage for the next steps (Figure 2a). Scratch assay confirmed that 50 ng/μl L‐HFS promoted cell migration of keratinocytes and fibroblasts when compared to the control group which is the normal culture condition without the presence of L‐HFS (Figure 2b,c). Especially in the human keratinocytes, L‐HFS treatment had led to complete healing of the scratch at 48 h while the scratch gap remained open in the control group. Myofibroblast contraction is a crucial event that controls scar formation. We used the fibroblast populated collagen lattice (FPCL) model to mimic fibroblast contraction. After incubating with L‐HFS for 7 days, shrinkage of the collagen lattice was suppressed, and the surface area of the L‐HFS treated lattice was significantly higher than that of the control (normal culture without L‐HFS) group (Figure 2d,e). The collagen lattice was subject to paraffin section and H&E staining to demonstrate the structure of FPCL. Same with the gross view, the staining result indicated that the L‐HFS treatment inhibits cell contraction. We also estimated the types I and III collagen accumulation in FPCL slides using picrosirius‐red staining and polarized microscopy examination. The L‐HFS treatment group had less type I collagen and more type III collagen (Figure 2g,h). Procollagen type III is the precursor of collagen type III. We have also found the expression of pro‐collagen type III in the L‐HFS treated group was significantly decreased, suggesting that L‐HFS treatment promotes type III collagen maturation. More pro‐collagen type III has been transformed into collagen type III in the L‐HFS group (Figure 2g,i). The fetal skin cell produces more type III collagen than the adult skin cell. The L‐HFS treatment may suppress the scar formation and increase the ratio of type III/type I collagen in the adult skin cell. These results suggest that L‐HFS may turn the adult cell into fetal cell status.

### 2.3 HFS encapsulated in PLGA particles retained bioactivities of HFS in vitro

**FIGURE 3:**
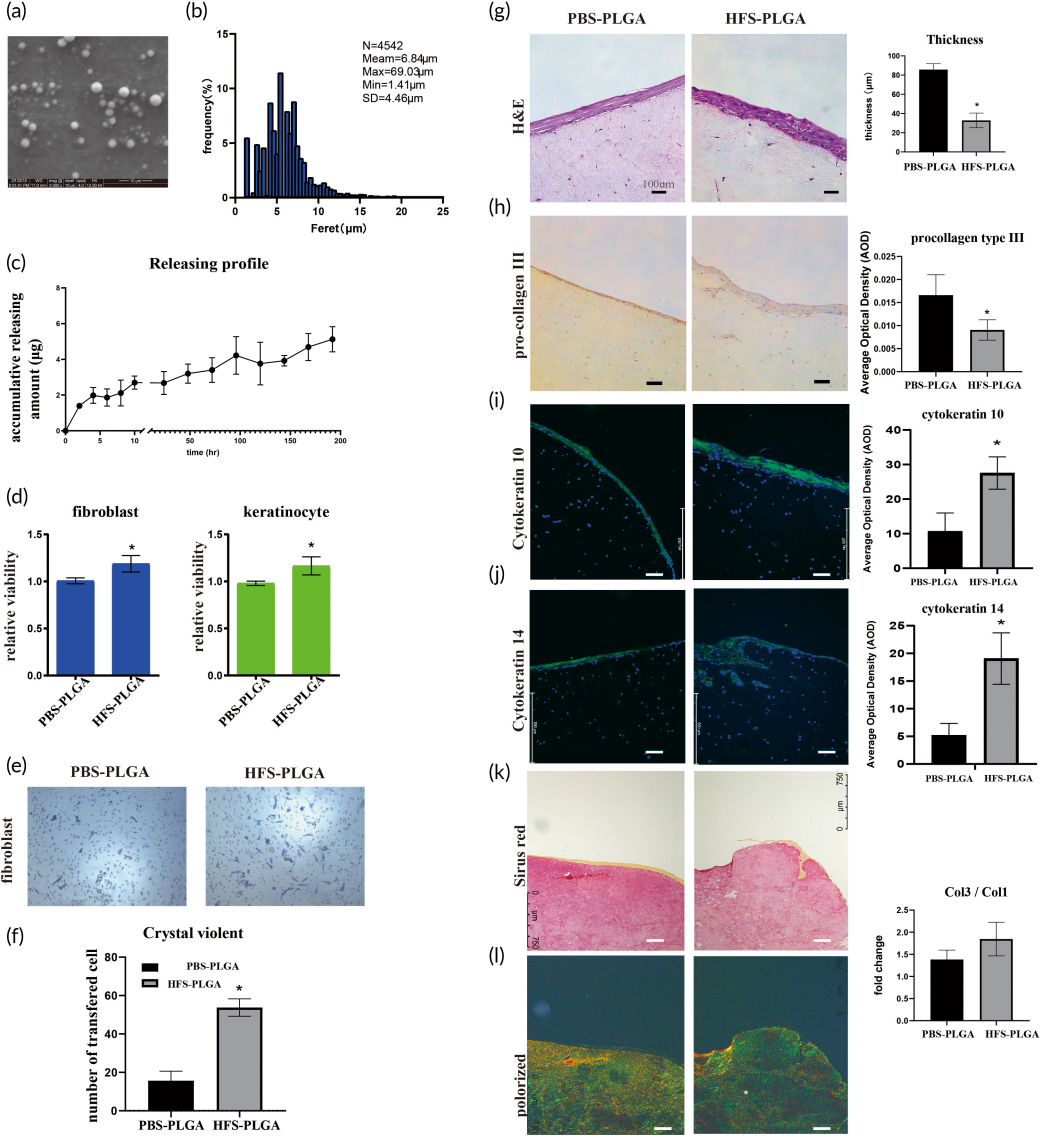
Human fetal secretome‐poly lactic‐*co*‐glycolic acid (HFS‐PLGA) retains the beneficial effect of HFS on human skin cells. (a). Transmission electron scanning microscopy showed spherical morphology of PLGA particles. The average diameter of the HFS‐PLGA sphere is around 10 μm. (b) Size distribution is measured with ImageJ based on the microscope picture of PLGA particles. The mean diameter of the particles is 6.84 μm. (c). The accumulative releasing curve of HFS‐PLGA showed that the HFS was kept releasing from PLGA particles up to 8 days. (d) Alamar blue results indicated that HFS‐PLGA could promote human skin cells' proliferation ability in trans‐well; (e) Crystal violet staining showed that HFS‐PLGA could attract human fibroblast during in the co‐culture system; (f) Quantitative result of crystal violet staining. Crystal violet were dissolved in 33% acetate and absorbance was measured at 570 nm. HFS‐PLGA group showed significantly higher absorbance compare to the PBS‐PLGA group. *N* = 3; (g) Represented view of staining result of KOC slides and quantitative result. H&E staining showed that HFS‐PLGA increased the thickness of the keratinocyte layer in KOC. (h). Immuno‐staining showed that HFS‐PLGA decreased pro‐collagen type III staining. The quantitative data was measured by image J; I‐J. Immuno‐fluorescence staining showed that HFS‐PLGA increase the expression of cytokeratin 10 and cytokeratin 14 in KOC model. The quantitative data were measured by image J; K. Sirius red staining and polarized microscopy examination showed a higher ratio of collagen III to collagen I in HFS‐PLGA group. The quantitative data were measured by image J based on the polarized signaling, green color represents type III collagen and yellow color represents type I collagen; Statistic: Mann–Whitney *U* (unpaired, nonparametric *T*‐tests). *N* = 3, *p < 0.5. Brightness and contrast were adjusted for clear demonstrated contents of the representative pictures

In the wound area, direct application of HFS powder cannot retain its bioactivities for long due to blood flushing or enzyme degradation. We used PLGA particles to encapsulate L‐HFS powder (HFS‐PLGA). The transmission electron microscopy (TEM) examination demonstrated the spherical morphology of the particles (Figure 3a). We have measured the particle size based on the microscopy pictures. The diameter and number of particles were measured by ImageJ. A total of 4542 particles are included in the statistics. The mean diameter of HFS‐PLGA particles is 6.84 μm. Min diameter is 1.41 μm, Max diameter is 69.03 μm. The encapsulation efficiency of PLGA is 19.5% (Figure 3b). The accumulative release curve of the protein concentration in PBS indicated that HFS‐PLGA kept releasing HFS for 8 days (Figure 3c), and the HFS was gradually released along with the PLGA shell dissolving. We also used a trans‐well system to test the effects of HFS‐PLGA in primary cultured human fibroblast and keratinocyte. After 3 days co‐culture, Alamar blue test showed HFS‐PLGA promoted cell proliferation of fibroblast and keratinocyte (Figure 3d), and HFS‐PLGA attracted significantly more fibroblasts migration compared to the PLGA‐PBS group while we fail to observe the attraction effect of HFS‐PLGA to the human keratinocyte (Figure 3e,f). The keratinocyte organotypic culture (KOC) model mimics the double‐layer structure of the skin. In the KOC model, HFS‐PLGA treatment significantly increased the thickness of the keratinocyte layer (Figure 3g). We also found the cornified pearl in the keratinocyte layer in the HFS‐PLGA group. This result suggested that HFS‐PLGA promoted keratinocyte maturation compared to the control group. Immunohistochemistry staining showed that the HFS‐PLGA group had reduced expression of type III pro‐collagen (Figure 3h). We use cytokeratin 10 (CK10) and cytokeratin 14 (CK14) as two proteins marker of keratinocyte maturation. CK10 is localized in the suprabasal layer and superficial layer of the epidermis of fetal skin.^33^ CK14 is localized in the basal layer to interact with skin stem cells corresponding to the proliferation and differentiation of keratinocytes.^34^ We performed the IF staining on KOC slides of CK14 and CK10 to indicate the maturation process of human keratinocytes (Figure 3i,j). The quantitative result shows that CK14 and CK10 are highly expressed in the PLGA‐HFS group. The proliferation and differentiation of keratinocytes are activated after our treatment. Same with the FPCL model, HFS‐PLGA treatment increases the ratio of collagen type III to collagen type I after Sirius red staining, indicating that PLGA particles preserve the anti‐aging effect of HFS(Figure 3k,l).

### 2.4 HFS‐PLGA particles promoted dermal repair in STZ‐induced diabetes rats

**FIGURE 4:**
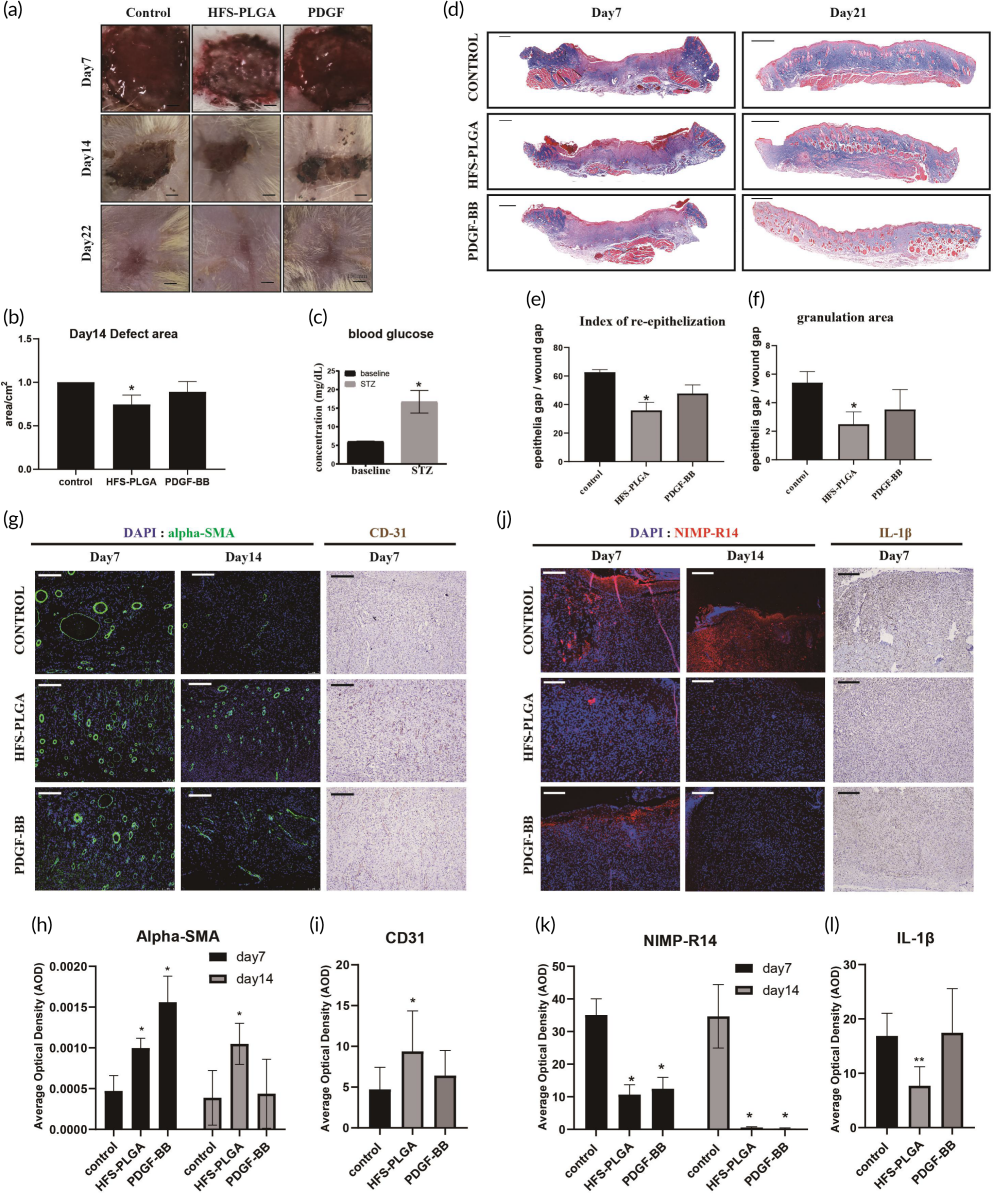
human fetal secretome‐poly lactic‐*co*‐glycolic acid (HFS‐PLGA) promote angiogenesis in the dermal injury of streptozotocin (STZ)‐induced diabetic Rat. (a) Representative picture of dermal injure at 7,14,21 days post‐surgery. Scalebar = 1.5 mm; (b) Semi‐quantification result of the wound area. HFS‐PLGA group significantly reduced wound area compared to the control group. The relative wound area of all groups was measured using image J. ANNOVA test, *N* = 4 *p < 0.05. (c) Higher blood glucose indicates that the STZ induction is successful. Mann–Whitney *U* *p < 0.05. (d) Representative picture of Masson trichrome staining on wound tissue sections in all time points. Scalebar = 1 mm; (e, f) Semi‐quantification data based on the color intensity of Masson trichrome staining. HFS‐PLGA treatment promoted re‐epithelization at Day 7 post‐surgery and reduced granulation area at Day 22 post‐surgery. Quantitative data were measured using image J. *N* = 4, Mann–Whitney *U* test, *p < 0.05; g Representative picture of immunofluorescent staining of Alpha‐SMA and immunohistochemistry staining result of CD31 result in the wound bed. Scalebar = 100 μm. (h,i) Semi‐quantitative result of alpha‐SMA and CD31 expression in the wound bed. HFS‐PLGA group significantly enhanced alpha‐SMA expression at Days 7 and 14 post‐surgery. CD31 were highly stained at Day 7 post‐surgery in HFS‐PLGA group. *N* = 4. ANNOVA test, *p < 0.05. (j) Representative picture of immunofluorescent staining of NIMP‐R14 and IL‐1βin the wound bed. HFS‐PLGA treatment suppress NIMP‐R14 and IL‐1βexpression at Day 7 post‐surgery. (J) The quantification results confirmed that HFS‐PLGA particles suppress the NIMP‐R14 and IL‐1βat the Days 7 and 14 post‐injury. *N* = 4. ANNOVA test, *p < 0.05. Brightness and contrast were adjusted for clear demonstrated contents of the representative pictures

Diabetic wound healing is impaired by high blood glucose. We then test the effect of HFS‐PLGA particles in vivo. After a one‐time high dosage i.p. injection, the STZ successfully elevated the blood glucose to 18 mg/ml while the control group only have 6 mg/ml (Figure 4c). In the STZ‐induced diabetic rats, standard skin wounds were made by surgery. HFS‐PLGA particles were applied by mixing with medical aqueous cream. HFS‐PLGA treatment significantly reduced the wound gap compared to the control group (PBS‐PLGA) 14 days after the wound creation (Figure 4a). Notably, the quantification result of the wound area indicated that the HFS‐PLGA group achieved a comparable effect as to the PDGF‐BB group (Figure. 4b). The skins of the wound area were then collected and subject to a paraffin section for staining. Masson trichrome showed represents the picture of the skin in each group at 7 and 21 days post‐surgery (Figure 4d). The quantification result showed that the HFS‐PLGA group had a longer epithelial layer (Figure 4e) and at 21 days post‐surgery, the HFS‐PLGA group had significantly less granulation area (Figure 4f). These results indicated that HFS‐PLGA accelerates the healing process in STZ‐Rat. Immunofluorescence results showed that both HFS‐PLGA and PDGF‐BB treatment groups enhanced the alpha‐SMA expression at 7 days post‐surgery, and the promoting effects on angiogenesis were only seen in the HFS‐PLGA group at 14 days post‐surgery. Immunohistochemistry staining showed that CD31 expression was significantly elevated after HFS‐PLGA treatment confirming that HFS‐PLGA has a pro‐angiogenesis effect (Figure 4g–i). We also tested the NIMP‐R14 expression at all time points. The immunofluorescence results showed that, at 7 and 14 days post‐surgery, HFS‐PLGA significantly suppress the neutrophils' migration to the wound bed. Immunohistochemistry results showed that IL‐1βexpression was suppressed by HFS‐PLGA treatment (Figure 4j–l). NIMP‐R14 is the neutrophil marker, the absence of the immune cell and the inflammation factors suggest that HFS‐PLGA suppressed the inflammation response in wound area.

### 2.5 Proteomics and bioinformatic analysis identified YWHAZ, HSPA8, and exosomes as candidate factors for pro‐angiogenesis effects of HFS

**FIGURE 5:**
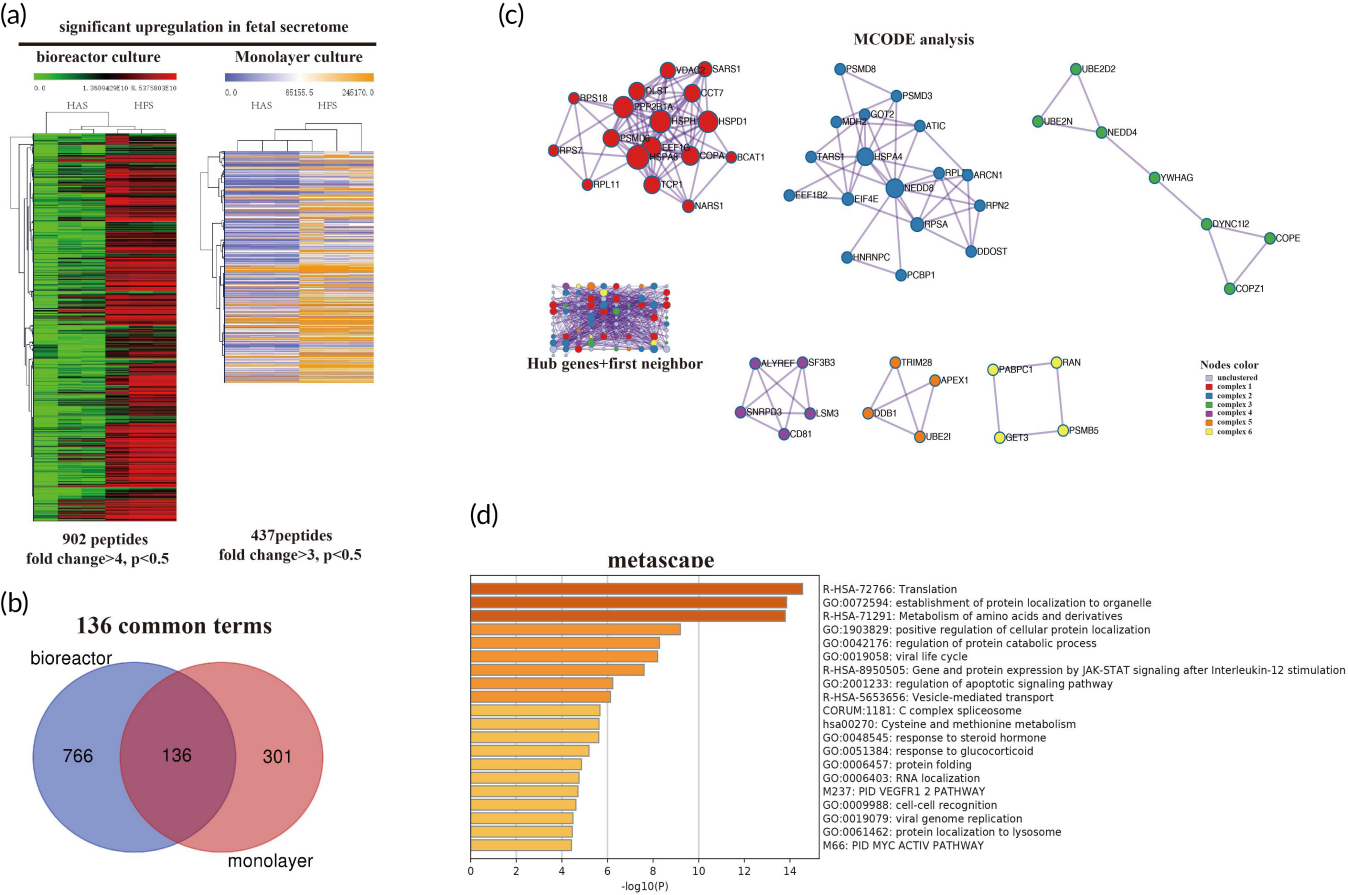
ITRAQ analysis revealed that the exosome, Heat‐shock proteins, and 14‐3‐3 proteins family play a core role in the fetal enriched proteins (FEP) network. (a). The expression heatmap of differentially expressed proteins (DEPs). The genes were pre‐selected base on Wilcoxon signed‐rank test. Only significantly highly expressed proteins (*p* < 0.05)were included in the heatmap. Rows represent individual proteins identified and relatively quantified by iTRAQ. Columns represent the individual samples. For bioreactor culture, the intensity scale is green, black and red. Green indicates lower and red higher expression in protein level. For monolayer culture, the intensity scale is blue, white, and yellow. Blue indicates lower expression and yellow higher expression of individual protein. The Rows and columns of the heatmaps are clustered based on Euclidean metric. Secretome from adult and fetal cells were successfully clustered together to each groups respectively. HAS: human adult secretome. HFS: human fetal secretome. Totally, 902 peptides and 437 peptides were identified as highly expressed protein in HFS. (b). The Venn diagram showing that 136 FEPs (fetal expressed proteins) were shared by bioreactor culture and monolayer culture. (c). A hub network was constructed by Metascape, with default parameters, based on PPI (protein–protein interaction) data extracted from STRING. (Left‐lower panel), MCODE in Metascape analysis identified six complexes from this network. Red, blue, green, purple, brown, and yellow color represent various sub‐networks. The knots in each subnetwork represent genes while the line between them indicate physical interaction. The size of the nodes indicates the degree of freedom of each gene. The Larger nodes indicates more genes were linked to this node. Hub genes: genes consist of complexes 1–6. First neighbor: other genes in 136 shared genes except the hub genes. (d) GO (gene ortholog) enrichment analysis was used to identify the biological meaning of the hub network. The statistically differentially expressed pathway was listed based on significant ranking. The Darker brown color represents the higher significance of each term. The bars represent the significance of the terms.

**FIGURE 6:**
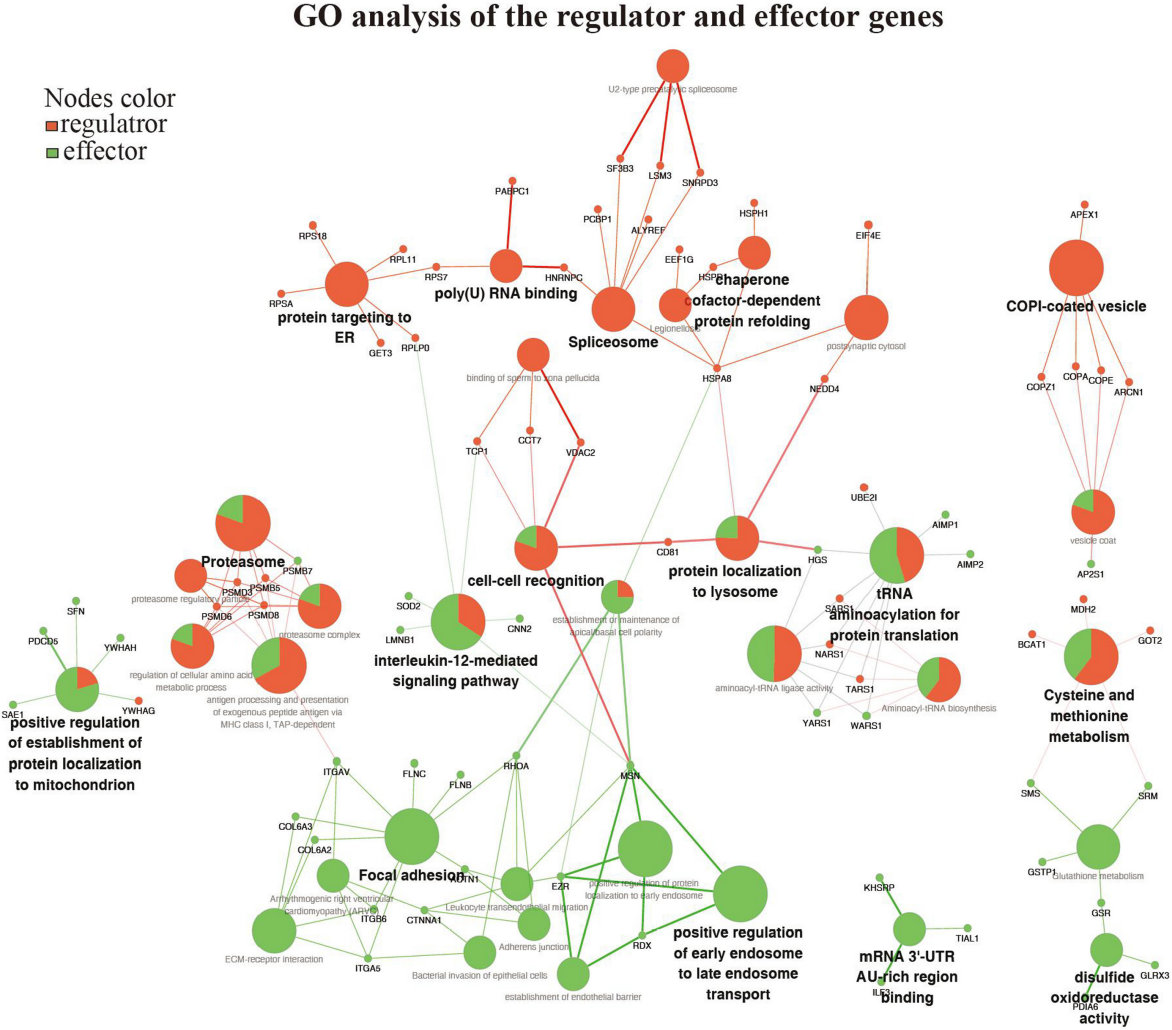
Gene ortholog (GO) network of regulator and effector genes. (a) ClueGO analysis was applied to the regulators and the effectors genes, respectively. The GO terms were shown as nodes and linked based on the kappa score (≥0.3). Only the most significant proteins enriched in each term were shown in the plots. The node size represents the enrichment significance. The red color represents the regulator proteins, the green color represents the effector proteins. The proportions of the two types of genes in mixed terms was visited as green and red color in one node. The distribution of two groups visualized on network based on their kappa score level (≥0.3), where only the label of the most significant term per group is shown as the smallest nodes. The larger node size represents the enrichment significance of this term

**FIGURE 7:**
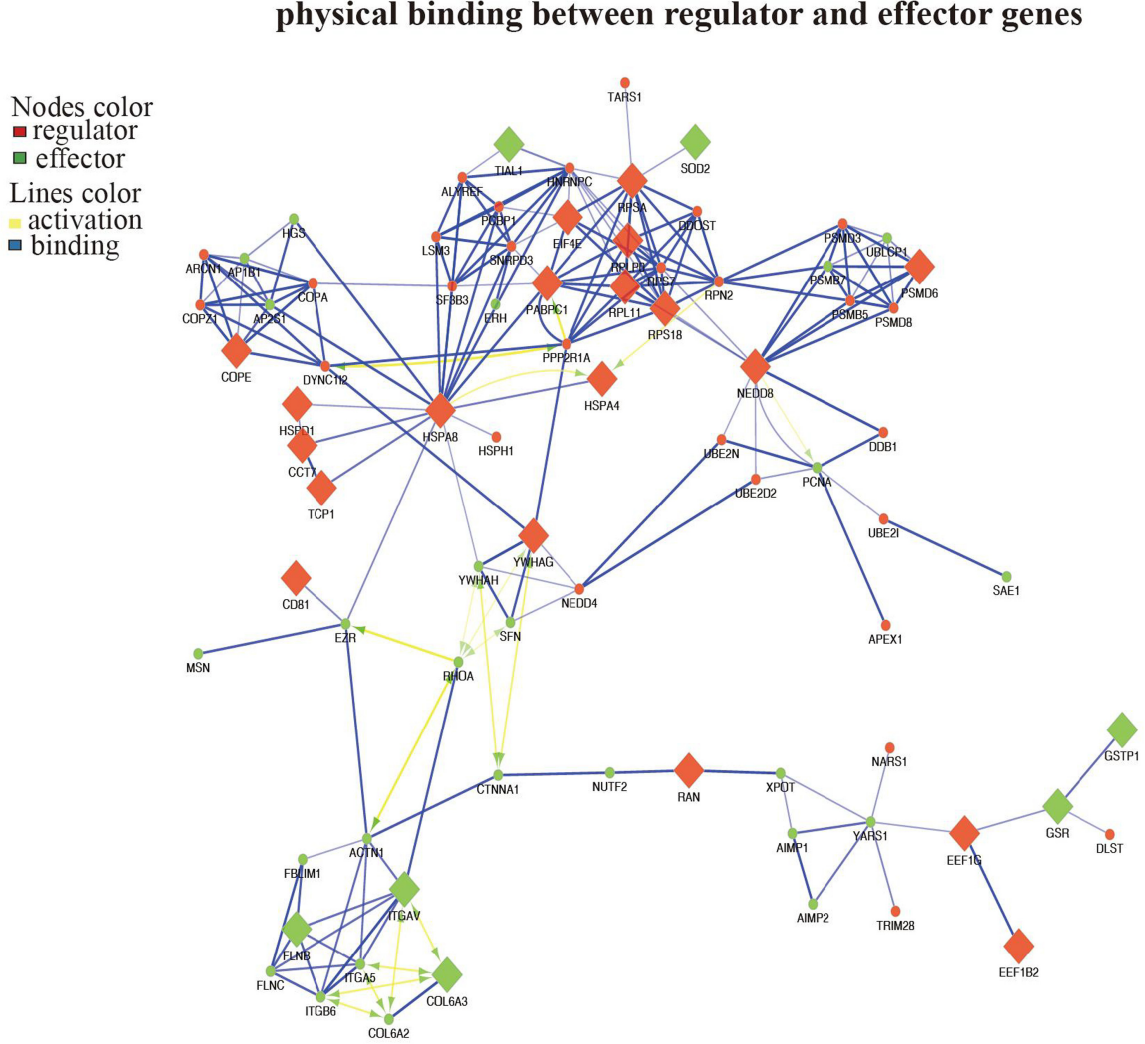
Physical network between regulator and effector genes. (a) Binding network was formed based on the gene ortholog (GO) functional network. Only the proteins have binding with each other are shown in the network. The blue line represents the physical binding between the proteins. The yellow line with the green arrow represents the positive regulation among the proteins. The plots were generated by Cytoscape with yfiles organic layout. The regulators and the effector were labeled with red and green colors

Our in vivo and in vitro studies have demonstrated that the HFS does have unique factors that have rejuvenating effects. To establish a functional biomarker of the secretome, we employed high‐throughput methods (ITRAQ and label‐free) to identify the exact protein that contained in the secretome. Highly differentially expressed proteins (DEPs) were identified by comparing HFS and HAS which is shown in Figure 5a. The ITRAQ method has been used for protein identification in our previous study for monolayer cultured secretomes. In the present study, we use a label‐free method to identify the protein in the secretome derived from bioreactor culture. Two datasets were combined to remove the influence caused by the change of the culture style (monolayer vs. bioreactor). Seven‐hundred sixty six proteins were upregulated in the bioreactor culture and 301 in the monolayer culture in the HFS (vs. HAS), and 136 shared proteins, named as FEPs (fetal enriched proteins), were selected for further analysis (Figure 5b). Based on FEPs, a protein–protein interaction (PPI) network was constructed in cytoscape. The interaction information was extracted from the STRING database. We then used CytoHubba, a plugin of cytoscape, to identify hub proteins. A hub network was established by combining the hub proteins and their first‐neighbor proteins. All the proteins within the hub network were subject to Metascape for visualization and further analysis. We identified six undestroyable sub‐network within the hub network. The sub‐networks, also known as MCODE complexes, were labeled with various colors while the other proteins were labeled with purple (Figure 5c). The MCODE complexes were regarded as the core components of the hub network. The Metascape also performed GO enrichment based on hub‐network, the proteins were enriched on several biological processes (BPs) in the HFS (Figure 5d). Among which VEGFR1 pathway was highlighted supporting that HFS has strong pro‐angiogenic properties. We further performed GO enrichment based on the regulators and the effectors in ClueGo (Figure 5e). The enriched terms were divided into three categories. The first category was only enriched with the regulators such as spliceosome, proteins targeting ER, and COVI‐coated vehicles. The second category was co‐enriched with the regulators and the effectors, including proteins in mitochondria, lysosome, and tRNA aminoacylation for protein translation, responsible for signaling transduction from the nucleus event to the cellular level. The third category is only enriched by the effectors, two terms related to focal adhesion and endosome transport were highlighted. FEPs enriched on the abovementioned terms suggested that hfMSCs had higher activities in cell proliferation, migration, extracellular secretion, and protein synthesis and degradation compared to those of haMSCs. The GO results suggested that exosome secretion and protein degradation are the two crucial properties of HFS (better) biological function (Figure 6). A physical network was constructed based on the physical binding among proteins of the regulators and the effectors. Several proteins were shared by the regulators and the effectors, such as HSPA8, HSPA4, YWHAG, and YWHAH. These proteins might be responsible for the functions of the whole network since they are physically binding to most of the complexes (Figure 7). Most of those proteins belonged to the second category in the GO analysis result, and the regulators might control the effectors through these switch proteins. In summary, the bioinformatic result revealed that the exosomes, 14–3‐3 family proteins, and heat‐shock proteins might be the main contributors to the biological functions of the HFS, and they might be potential biomarkers for quality controls of HFS production.

### 2.6 Intracellular exosomes, YWHAH, and HSPA8 regulate angiogenesis

**FIGURE 8:**
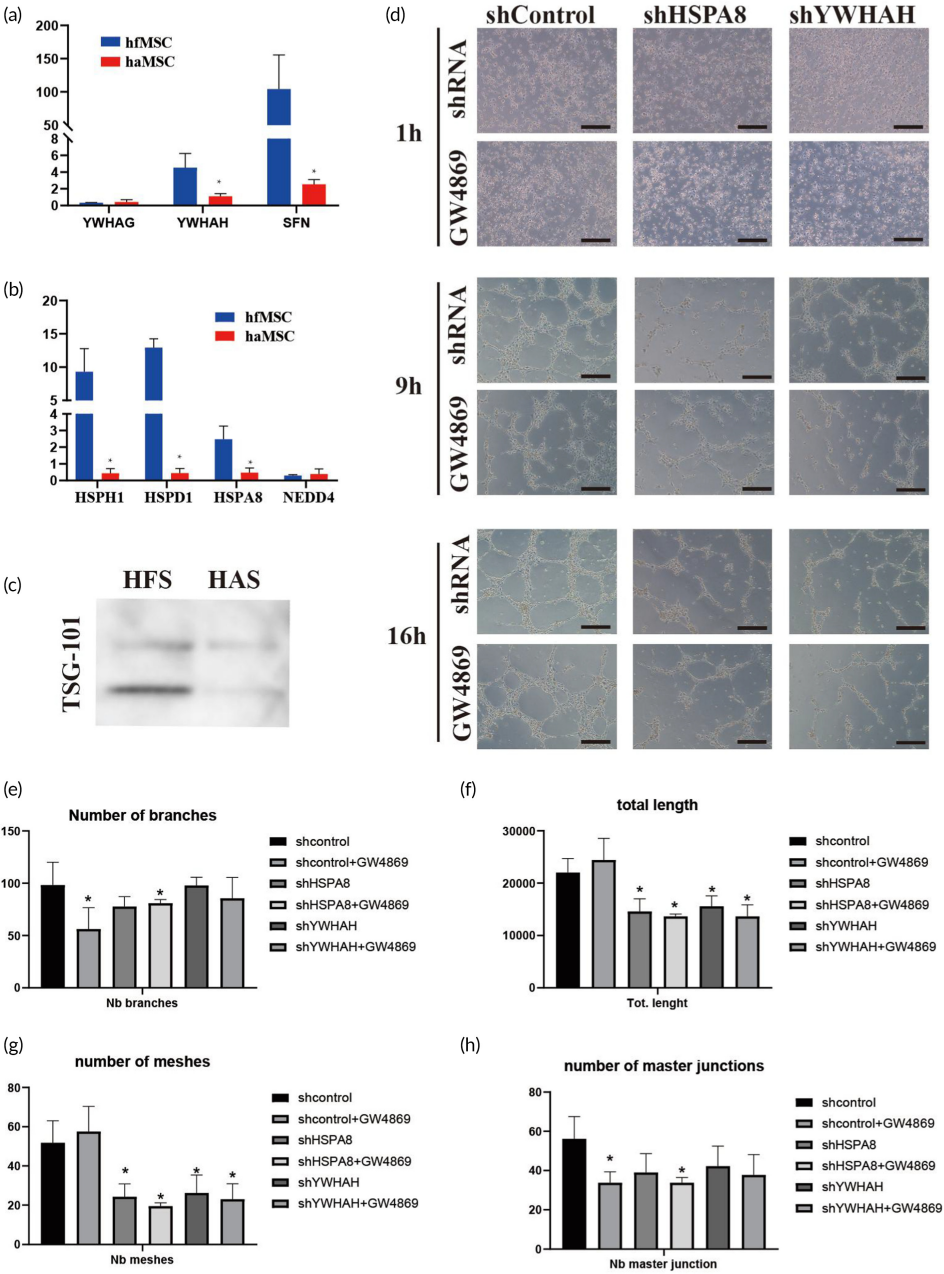
Exosome, YWHAH, and HSPA8 controlled the angiogenesis in tube formation assay. (a,b) QPCR validation of the candidate proteins. A 14‐3‐3 family and heat‐shock protein are highly expressed in human fetal mesenchymal stem cells (hfMSC) comparing to HAMSC. (c) Western blot result showed that TSG‐101 is highly contained in human umbilical vein endothelial cells (HFS) comparing with HAS. (d) Representative picture of tube formation of human umbilical vein endothelial cell (HUVEC) in the trans‐well system. Data were taken from three time points, 1, 9, and 16 h. Knockdown of exosome, HSPA8, and YWHAH in 293T cell reduced tube‐formation of HUVEC. (e–h) Quantitative result of tube formation indexes. The angiogenesis analyzer® plugin in Image J was used for measurement. The number of branches, total length, number of meshes, and number of masters junctions were measured based on the picture of tube formation. Statistic: Mann–Whitney *U* (unpaired, nonparametric *T*‐tests). *N* = 4, *p < 0.5

We performed quantitative polymerase chain reaction (qPCR) to validate the expression of the candidate proteins selected from the bioinformatic analysis. The results showed that YWHAG, SFN, YWHAH, HSPH1, HSPD1, HSPA8, and NEDD4 were highly expressed in hfMSCs (Figure 8a,b) compared to haMSCs. We also performed a western blot to confirm the high expression of TSG‐101 in HFS, an exosome biomarker, indicating that HFS contained more exosomes (Figure 8c). We performed a tube formation assay to verify the effects of secreted exosomes and proteins on blood vessel formation. The shRNA plasmids of HSPA8 and YWHAH were constructed and transferred into 293T cells and the gene knocking down 293T cells were co‐cultured with human umbilical vein endothelial cells (HUVECs) in a trans‐well culture system. GW‐4869, an exosome biogenesis inhibitor, was used to knock down the production of exosomes in cells. The tube formation assay results showed that inhibition of cell‐secreted exosomes reduced tube formation of HUVECs in both shRNA and GW‐4869 treatment groups. GW‐4869 treatment significantly decreased the number of master junctions and the number of branches, while the total length and number of meshes were not affected, suggesting that the exosomes mainly regulated the migration and proliferation of HUVECs. The sh‐YWHAH and sh‐HSPA8 groups decreased the values of all four indexes, indicating that intracellular YWHAH and HSPA8 also controlled the elongation and tube formation, and angiogenesis process (Figure 8e–h). When GW‐4869 and shRNAs were applied to the cells together, no additional inhibitory effects on angiogenesis were found, suggesting that HSPA8 and YWHAH might exert their functions independently in the exosome (Figure 8d,e). These results indicated that the exosomes, HSPA8, and YWHAH in the HFS might be the angiogenic regulators, and the soluble HSPA8 and YWHAH might be used as biomarkers for quality control for HFS production.

## 3 DISCUSSION

The present study used bioreactors and microcarriers to culture expand the hfMSC on large scale. HFS is lyophilized for long‐term storage, easy to transport and use. The L‐HFS were encapsulated into PLGA particles for slow‐releasing purposes, and HFS‐PLGA significantly promoted wound healing in STZ‐induced diabetic rats. Thus, our study successfully developed a bioproduct that might facilitate the treatment of diabetic foot ulcers. PDGF‐BB is an FDA‐approved biological drug for wound healing management, it promotes healing through stimulating granulation tissue formation, enhancing re‐epithelialization and vascularization as well as promoting collagen production.^35^ In the present study, we found that HFS exerted comparable if not better effects than PDGF‐BB. Cell source, in vitro expansion, and quality control, are three major aspects that decide whether the mesenchymal stem cell is suitable to be an Off‐the‐shelf bioproduct. Our study tried to prove several new concepts focusing on improving the production of stem cell secretome, which is a new direction of stem cell therapy. The MSCs have been isolated from various tissues, bone marrow,^36^ adipose tissue,^37^ and umbilical cord^38^ are most used for cell isolation. These adult tissue‐derived MSC have a common bottleneck, the cellular senescence limiting the cell expansion. The long‐term and low efficiency of the in vitro expansion reduced the production rate of stem cell secretome. The human fetal mesenchymal stem cell we used in the present study was isolated from the bone marrow of a human fetus. Compared with the three commonly used MSC, hfMSC was more proliferative and potency. Studying the properties of the fetal cell might provide new clues to improve the anti‐senescence capabilities of the adult MSC in the future. Our study proved that in the bioreactor culture, fetal cells produced more regenerative factors, such as PDGF‐BB and VEGF compared with the adult MSC. The hfMSC produced lactate is significantly lower than that those of haMSC. A high concentration of lactate in the culture medium indicates the cell metabolic status of the adult MSC is close to the aerobic glycolysis.^39^ The fetal cell may tend to use oxidative phosphorylation pathway as a major energy supply. This was supported by our previous study that transferred fetal cell mitochondria triggered metabolic reprogramming of the adult cell.^10^ The metabolic status of the MSC might correlate with cellular senescence. Less acidified microenvironments might play an important role in promoting in vitro expansion.

The fetal cell properties are not only beneficial for cell proliferation. It is also important for tissue repair in terms of scarless healing in skin wounds. One crucial event during skin wound healing is fibroblast‐myoblast transformation. Myoblast's contraction helps wound closure. However, excessive myoblasts contraction could lead to scar formation.^40^ Contractures limit the normal growth of skin cells and impede the fully functional recovery of skin wounds. Scarless healing in the early gestation stage in mammals was reported in 1971, and the intrinsic properties of the fetal cells mainly contribute to the anti‐scar ability.^41^ It has been known that fetal skin mainly consists of type III collagen while adult skin mainly consists of type I collagen. Excessive deposition of type I contributes to scar formation. A high ratio of Type III to Type I collagen indicates better anti‐contraction potential.^42^ Our data have demonstrated that HFS treatment inhibited fibroblast contraction and increased the ratio of typeIII/type I collagen. This result suggests that HFS might endow some fetal cell properties to the adult skin cell.

There are two major types of bioreactors that have been used to scale up the MSC expansion. The hollow fiber‐based system^43^ and microcarrier‐based system.^44^ These two systems both have been used for the culture of various types of MSC. Compared with monolayer culture, bioreactor culture could produce 15–20 fold of the cell using the same volume of the culture medium. The stem cell properties could be maintained during the large‐scale expansion.^45^ The hollow fiber system is static culture while the microcarrier system requires the stirred tank to maintain the refreshment of oxygen and nutrition. In our study, we choose the vertical stirred tank to scale up the hfMSC since the tank is more convenient to collect the secretome as more as possible. The culture medium did not need to be refreshed too often during the culture period.

The microcarrier system is more widely accepted than the hollow fiber system. Our present study chooses a small bioreactor with a 100 ml culture volume. We proved the feasibility of expanding MSC in the bioreactor. The secretome we collected under this culture condition kept its regenerative function unchanged. The bioreactor‐derived HFS showed a similar rejuvenating effect as the secretome derived from monolayer culture. A recent study has scaled up the MSC culture in a 50‐L bioreactor using a microcarrier system.^46^ We may scale up the current system in the future.

The safety and effectiveness of MSCs therapy are still under debate, there are concerns that injection of cells, as well as cellular components, may induce side effects, such as immune responses and risks of infection and disease transmission. It is well accepted that MSCs culture conditioned media contain many biological active factors is responsible for modulation of inflammation, cell recruitment, matrix deposition, and cell proliferation.^47^ MSCs could release anti‐inflammatory factors such as IL‐4, ‐6 and ‐10, and TGF‐β.^48^, ^49^ Furthermore, MSCs stimulate the proliferation and migration of various types of cells involved in wound healing via paracrine factors.^50^ Thus, MSCs‐derived secretory factors could be a reliable sources for promoting wound healing. Moreover, our study analyzed the protein content in HFS through proteomics and bioinformatic analysis. We had identified several key signaling pathways that might contribute to the unique properties of hfMSC. Among these, heat‐shock proteins and 14‐3‐3 proteins were identified as two critical families corresponding to multiple cellular functions. We found that HSPA8 and YWHAH, unique contents of HFS, significantly promote angiogenesis, were crucial in wound healing. We also found multiple biological processes correlate with the biogenesis of exosomes in hfMSC. The 14‐3‐3 protein family was also regarded as a sub‐family of heat‐shock protein. Usually, they exert their function together with the other heat‐shock proteins to protect the cell from physiological stress.^51^, ^52^ The crucial role of YWHAH and HSPA8 in our protein network indicated that heat‐shock protein might play an important role in the superior role of hfMSC. Furthermore, the heat‐shock protein and cellular senescence were closely correlated with each other. The fetal cells might have a unique cellular defensive mechanism helping to fight against senescence.

Collectively, our study elucidated novel factors regulating angiogenesis from the hfMSC secretome. The presence of exosomes, HSPA8, and YWHAH might be taken as unique biomarkers for quality control of HFS, this is important for the standardization of production of MSC secretome production.

Stem cell secretome is a mixture, current studies focused on verifying its biological functions. The mechanisms behind these functions remained largely unknown, which limits the wider clinical applications of MSCs secretome. One possible research direction is to analyze the bioactive contents of the secretome in detail. In the present study, we used a high‐throughput proteomics technique and determined that the main components of secretomes are exosomes. Exomere is a newly founded subclass of exosomes, that mainly contains proteins related to the glycolysis pathway and mTORC pathway, suggesting that exomere is involved in regulating energy metabolism and cell proliferation.^53^ To date, the biological functions of exomere derived from mesenchymal stem cells are unknown and there are no universal biomarkers for exomere. The role of exomere in tissue repair is interesting. One important finding of our study was that we confirmed that HSPA8 mediated wound healing. In HFS, we had identified that HSPA8 was highly expressed, which had also been founded in the tumor as exomere.^53^ Thus, HFS contains exomere that might regulate cell energy metabolism and other activities.

The Warburg effect is a well‐known phenomenon in that cells tend to utilize the tricarboxylic acid (TCA) pathway for energy metabolism under physiological conditions and switch to anaerobic glycolysis when they are under stress such as in tumorigenesis and anaerobic glycolysis is regarded as a more efficient manner to use the limited ATP.^54^ The production of lactate reflects the activity of glycolysis. Our ITRAQ analysis indicated that HFS contained factors that regulated glycolysis such as HSPA8. However, hfMSCs produced extremely lower lactate in bioreactor culture compared to the haMSCs, suggesting that hfMSCs had unique and superior metabolic properties. Recently, studies reported that glycolysis activity was up‐regulated during tissue repair^55^ and the glycolysis signals released from the tumor cells stimulated the growth of surrounding mesenchymal tissues.^56^ Tissue repair and tumor growth‐share many common signaling pathways such as angiogenesis and energy consumption. Topical application of HFS may directly regulate glycolysis and promote angiogenesis during wound healing. Therefore, targeting the glycolysis pathway may be a new therapeutic strategy for promoting tissue regeneration.

Stem cell secretome has the potential to be industrialized. However, our production platform is still in its imperfect. Our study is currently limited to the laboratory. A larger scale is needed to meet the requirements of industrialization. We need to enlarge the culture volume from 100 ml to 3–5 L. A larger scale of culture not only requires increasing the input of culture materials but also requires the real‐time monitoring of CO_2_ and other cell physiological indexes. An advanced culture platform is needed. The cell source is another obstacle to be overcome. In our study, we used human fetal MSCs as the source of secretome due to their unique properties. A high proliferation rate allows hfMSC to be sufficiently expanded on a large scale while haMSC failed to do so. However, hfMSCs have ethical issues in commercial applications. Human umbilical stem cells (huMSCs) might be a suitable replacer for the production of the secretome on a large scale.^57^ As a clinical waste, huMSCs have no ethical issue. Especially, huMSC is derived from post‐natal tissue, the same with hfMSC, huMSC is derived from a relatively earlier developmental stage compared with haMSC. A comparable regenerative property is expectable in huMSCs secretome. Genetically modified cell lines might be another solution to bypass the ethical issue. CRISPR systems have been invented and draw wide attention with a huge potential in translation medicine. U.S. FDA has approved using the CRISPR‐based method for rapid detection as well as the clinical trial for the treatment of rare diseases.^58^, ^59^

## 4 MATERIALS AND METHODS

### 4.1 Isolation and characterization of hfMSCs

Human fetal bone tissues obtained from surgical termination of pregnancy (joint university‐hospital ethics committee approval code: CREC‐2011.383 and patient's full consent) were harvested for hfMSCs isolation. The fetal limbs as clinical waste were collected in the surgical room, transported to the cleanroom (GMP standard), and processed for the isolation of hfMSCs as previously described.^11^ hfMSCs are cultured in Dulbecco's modified Eagle's medium (DMEM) supplemented with 10% fetal bovine serum (FBS) and 1% GlutaMax (Life Technologies, Grand Island, NY, USA). Expanded hfMSCs are subject to trilineage differentiation and flow cytometry for surface marker detection. All the MSCs were used before passage 5 to ensure the cells retain sufficient stemness.

**Large‐scale production of hfMSCs secretion (HFS).** The current protocol for harvesting MSCs secretome from monolayer culture is time‐ and labor‐consuming which cannot meet the clinical need for large‐scale production of HFS. To enhance productivity, a vertical bioreactor is employed according to the previously reported methods.^60^ In brief, hfMSCs are pre‐cultured in normal layer culture under the normal culture condition. Until the cell number meets the requirement. The cells were seeded on collagen‐coated polystyrene microcarriers (Solohill). The cells and micorcarriers were premixed and waited for 2 h before the agitation started. A 100 ml complete alpha‐minimum essential medium (α‐MEM) containing 10% FBS and 1% antibiotics was used to continue culture the cells during the bioreactor. The optimal culture condition is 1 million cells/3 g microcarrier in a 100 ml culture medium, After 2 weeks of culture, the cells‐microcarriers a serum freeα‐MEM for 24 h in the bioreactor system (PBS Mini, Vertical‐Wheel™ Bioreactors, 1183 Calle Suerte, Camarillo, California 93,012, USA). The conditioned medium (CM) is then collected, and residual cells and micro‐carriers are removed by centrifugation at 1000*g* for 5 min at 4°C. The supernatant is concentrated by lyophilization. The protein concentration of concentrated CM, named HFS, is determined using Pierce™ BCA protein assay kit (Life Technologies, USA) at 562 nm as in‐house quality control. The concentrations of glucose, ammonia, lactate, and pH in the bioreactor are monitored every 2 days to ensure healthy cell growth. The optimal seeding density of hfMSCs is determined with an Alarmar blue (Invitrogen, USA) test and two‐color fluorescence EthD‐1/Calcian AM live/dead assay as per the manufacturer's instructions. Alamar blue is used to measure the cell proliferation ability,

We have calculated the cell number before and after plating at two‐time points, 5‐ and 10‐days post cell seeding. At each stage, samples were collected for quantification. In brief, 5 ml of well‐mixed cell‐laden microcarrier suspension was transferred into 15 ml sample tubes and allowed to settle. Samples were then washed twice with 5 ml PBS to remove the residue medium. After discarding the supernatant, 2 ml 0.25% Trypsin–EDTA was added to the microcarriers to allow the cells to detach from the microcarriers. 10uL cell suspension was then applied to the hemocytometer.

### 4.2 Protein identification of secretome from a fetal and adult cell

All proteomics analyses were sent to BGI (Shenzhen, China). Three biological replicates were carried out for each sample. We used the previously described^61^ ITRAQ method to identify proteins for monolayer cultured secretome. Briefly, the HFS will be precipitated by acetone at −20°C overnight to obtain a reduced and alkylated protein mixture. A 100 μg of each sample solution is digested with Trypsin Gold (Promega, Madison, WI, USA). After digestion, peptides are labeled with an 8‐plex iTRAQ reagent (Applied Biosystems, USA) according to the manufacturer's protocol. An LC‐20AB HPLC pump system (Shimadzu, Kyoto, Japan) was used to separate each sample into 20 fractions. Each fraction was re‐suspended, then the peptides are subjected to nanoelectrospray ionization followed by tandem mass spectrometry (MS/MS) in a QEXACTIVE (Thermo Fisher Scientific, San Jose, CA, USA) coupled online to the HPLC. Raw data files acquired from the Orbitrap will be converted into MGF files using Proteome Discoverer 1.2 (PD 1.2, Thermo‐Fisher, USA). Protein identifications will be performed by using Maxquant with a search engine. For protein identification, the parameters followed the published study.^62^

We use a label‐free method to identify protein in the powder of L‐HFS. Due to protein concentration being reduced after reconstitution. It is no longer suitable for the ITRAQ procedure. BGI used a customized procedure to enhance the sensitivity of protein detection. Briefly, cold acetone was added to the L‐HFS solution at a ratio of 1:5, to precipitate the total protein in L‐HFS DTT was used to open the disulfide bond. IAM was used to block the cysteines. Coomassie blue and SDS‐PAGE were used to examine the degradation status. Subject samples to HPLC, 100 μg total proteins were subjected to HPLC with the pretreatment of trypsin. The mixture was subjected to Thermo Ultimate 3000 UHPLC with a trap column for desalted, and then entered a self‐packed C18 column and separated at a flow rate of 300 L/min with various concentrations of the liquid phase. The nanoliter liquid phase separation end was directly connected to the mass spectrometer (MS). In MS, peptides were ionized by a nanoESI source and then passed to a tandem mass spectrometer Q‐Exactive HFX (Thermo Fisher Scientific, San Jose, CA) for DDA (data‐dependent acquisition) mode detection. The ion fragmentation mode was HCD, and the fragment ions were detected in Orbitrap. The raw data from orbitrap will be processed to assemble the procedure of monolayer culture.

### 4.3 Bioinformatic analysis

For heatmap: Protein expression data from bioreactor culture and monolayer culture were extracted and subjected to Mev4.0.9 to generate the heatmap. We selected proteins with fold changes higher than four in bioreactor culture, and three times higher in monolayer culture as DEPs. DEPs in two culture methods were cross‐compared to identify the FEPs. The name of FEPs was converted to the official gene symbols using DAVID. They were subjected to Cytoscape to generate a scale‐free network. The linkage information was extracted from the STRING database. The hub proteins were calculated using Cytohubba with the default setting. The hub proteins and their first neighbors were extracted from the scale‐free network to construct a hub network. The proteins in hub‐work were submitted to Metascape for MCODE analysis and GO enrichment. The MCODE results were generated with the default setting in Medscape. The hub network was divided into the regulators and the effectors. The two groups of protein were submitted to ClueGO in Cytoscape separately to figure out the linkage between groups. Biological process terms were used to perform enrichment. The major perimeters are: kappa score = 0.3, Terms fusion = true, Cluepedia = true, files organic layout.

### 4.4 Preparation of HFS encapsulated in PLGA particles

The HFS were frozen at −80°C in the ultralow freezer for 6 h. The frozen samples were lyophilized in a freezer dryer (Labconco FreeZone Benchtop Freeze Dry System, Kansas City, MO, USA) for at least 48 h. HFS‐PLGA nanoparticles were prepared with the double‐emulsion technique as previously described.^63^ In brief, 200 μl of 2%–10% w/v HFS solution (dissolved with water) was mixed with 3.33 ml 3% w/v PLGA solution (dissolved with DCM, dichloromethane) and sonicated at 30 W for 2 min. This mixture was added dropwise to 12 ml 2% polyvinyl alcohol (PVA) solution and sonicated at 20 W for 2 min, and then de‐solvated by overnight stirring. Particle aggregates were removed by centrifugation at 4000 rpm for 5 min. The HFS encapsulated PLGA particles were washed and collected using ultracentrifugation and subject to lyophilization for prolonged storage.

The L‐HFS and PLGA were weighed at 0.1 and 2 g, respectively (weight ratio 1:20) to form HFS‐PLGA particles by double emulsion technique with lyophilization for 2 days. The optimized weight ratio is 1:20 in our present study. The percentage yield (the weight of lyophilized HFS‐PLGA/the weight of original material [L‐HFS + PLGA] × 100%) is 64%. The total amount of HFS‐PLGA is 2.1 g × 64% = 1.3 g. Then, 0.5 mg HFS‐PLGA particle preparation was suspended in 1.5 ml PBS solution (pH 7.4) and maintained at a 37°C incubator to obtain its releasing curve. Encapsulation efficiency was measured based on the previous study.^64^ Encapsulation efficiency (%) = amount of drug released from the lyophilized PLGA particles/amount of drug initially taken to prepare the particles × 100. According to the releasing curve, the HFS‐PLGA particle could release the HFS for up to 8 days. The final amount is 5 μg/ml. The total amount of HFS carried by 0.5 mg PLGA particles is 5 μg/ml × 1.5 ml = 7.5 μg. The encapsulation% = 7.5 μg × (1.3 g/0.5 mg)/0.1 g × 100% = 19.5%.

### 4.5 Cell proliferation and migration assays

The cell proliferation was determined with Alamar blue assay (Life Technologies, USA) as previously reported.^11^ Scratch assay was used for cell migration assay according to the previously described methods. The ability of HFS to recruit MSCs was also evaluated by transwell assay as previously described.^65^ In brief, haMSCs were seeded in transwell inserts at a density of 5 × 10^4^ cells/cm^2^ with 600‐μl serum‐free medium. The HFS at various concentrations were then added into the bottom chambers of the 24‐well plates. The cells were cultured for another 16 h, and residual cells on the upper surfaces of the inserts were removed with cotton swabs, while the migrated cells on the lower surfaces were stained with 0.5% crystal violet and counted.

### 4.6 Primary culture of human keratinocytes and fibroblast

Primary human keratinocytes were prepared as previously described with a few modifications.^66^ Normal human keratinocytes and fibroblasts harvested from pediatric foreskins were used. Briefly, the foreskin was surgically removed and cut into 1.0 × 0.5 cm strips, and incubated with trypsin/EDTA solution at 4°C overnight. The epidermis was then separated and keratinocytes were collected by centrifugation at 1400 rpm for 5 min and 2 × 10^6^ keratinocytes were seeded into a type IV collagen‐coated plate and cultured in complete K‐SFM medium (Gibco, USA) at 37°C.

For fibroblast culture, cells were prepared according to previous report.^67^ The dermis was mechanically separated from the epidermis. The dermis was digested with dispase overnight and finely minced. The resultant cell suspension was transferred to culture dishes and subculture in DMEM supplemented with 10% FBS. Cells were stored in liquid nitrogen.

### 4.7 Fibroblast‐populated collagen lattice

FPCL was prepared according to previously described protocols with minor modifications.^66^ In brief, six‐well plates were precoated with 1% agarose. Two milliliters of FPCLs containing 1.2 × 10^5^ cells and 1.25 mg/ml type I collagen in complete medium (DMEM supplemented with 10% FBS) were cast in the plates. The gels were then polymerized at 37°C for 30 min. Afterward, the gels were gently detached from the agarose surface to allow contraction, and 2 ml of complete media with or without L‐HFS was added per well.

### 4.8 Keratinocyte organotypic cultures

The organotypic 3D keratinocyte‐fibroblast co‐culture was prepared according to previously described protocols.^68^ A 5 mg/ml rat tail type I collagen mixed with 3 × DMEM and neutralized with 5 N NaOH to a final concentration of 3 mg/ml. A 600 μl each of this collagen solution was then loaded into the inserts (0.4 μm pore size) of the 12‐well transwell and incubated at 37°C for 2 h for gel polymerization. The insert was equilibrated with KGM overnight (keratinocyte growth medium), which is consists of a 1:3 mixture of Ham's F12 and DMEM, 10% FBS, 5 μg/ml insulin, 1.8 × 10^−4^ M adenine sulfate, 1 × 10^−10^ M cholera toxin, 0.4 μg/ml hydrocortisone, 1 ng/mL EGF and 0.1% BSA. KOC was created the next day after the FPCL was established. A 10 × 10 mm cell cylinder (Corning Incorporated, Acton, MA) was placed on top of FPCL to enable keratinocyte seeding. A total of 2 × 10^5^ keratinocytes suspended in 200 μl KGM were plated in the cylinder. After 24 h, the cylinder was removed and the insert was lifted to an air–liquid interface and cultured until used. HFS‐PLGA and PBS‐PLGA were added into the lower chamber from the day when the air‐lifted culture started.

### 4.9 Diabetic rat wound healing model

We have taken the gender effect into account when we designed the experiment. According to the literature, the sensitivity to STZ is significantly (*p* < 0.001) higher in male rats compared to female. The estrogen could protect pancreatic β cell from apoptosis induced by oxidative stress.^69^ We choose male rats in this study to increase the success rate of the STZ model. To establish a diabetic rat model, streptozotocin (STZ, Sigma‐Aldrich, USA) prepared in 0.1 M citrate buffer (pH 4.5) was injected intraperitoneally at the dose 50 mg/kg as described previously. STZ could induce diabetes within 3 days by destroying the β cells.^70^ The blood glucose was measured at 5 and 7 days after injection to confirm the onset of hyperglycemia. On day 10 following STZ injection the confirmed diabetic rats were used to create skin wounds according to the methods previously reported.^71^ In brief, under general anesthesia and sterile conditions, 4 circular wounds with a diameter of 8 mm and a depth of 2 mm were made on the back of a male SD rat (average bodyweight 250–280 g) with a punch biopsy device (Biopsy Punch, Miltex Inc., Pennsylvania 17,402, USA) on the upper back of each rat, and the skin flap was sutured with a round‐shaped silicone splint.

The HFS‐PLGA were applied topically at the wound site in aqueous cream. In each rat, the 4 holes were treated as the following: Hole 1: left untreated; Hole 2: covered by aqueous cream only; Hole 3: covered by low‐dose HFS encapsulated PLGA particles cream; Hole 4: covered by aqueous cream containing PDGF‐BB (1ug per wound). PDGF‐BB is used as a “positive” control in this study. A sterile dressing was used to cover the splint to allow the tissue and dressing to have no contact with each other. The dressing could prevent dehydration and contamination of the wound, the dressing was changed every 3 days.

### 4.10 Histological analysis and immunostaining examinations

All the control and treatment group pictures will be merged into one picture before starting. This step allows regulating two groups' parameters together to avoid objective bias.

We use the following steps to measure the A.O.D (average optical density).

1. The channel of the JPEG picture will be turned into RGB 8‐bit format.
2. Use the ROI tool to mark all the samples.
3. The threshold will be manually regulated based on one experience.
4. Set measurements‐tick the box: area / mean gray value / integrated density / limit to threshold.
5. Use the multi‐measure function to measure all the ROI together.
6. For each ROI, the mean fluorescence intensity = Integrated Density/area.

After 7, 14, and 28 days, animals (*n* = 4 per time point per group) were terminated, and the tissues at the wound area (8‐mm diameter including the complete epithelial margins) were harvested, and fixed in 4% paraformaldehyde for paraffin embedding. The degree of re‐epithelialization and granulation tissue formation were evaluated on histology sections by measuring the distance the epithelium across the wound; the muscle edges of the panniculus carnosus are used as an indicator for the wound margin; re‐epithelialization is calculated as the percentage of the distance of new epithelium covering the wound area. For granulation tissue quantification, the area covered by highly cellular tissues was determined and normalized with the distance of muscle edges of the panniculus carnosus. Paraffin sections were used for immunostaining examinations of inflammatory cells and blood vessels with antibodies against NIMP‐R14, IL‐1β，CD31, and alpha‐SMA, respectively. Alexa fluo‐conjugated or HRP‐conjugated secondary antibodies were used for fluorescent or IHC staining. The image were acquired with microscope (DM5500, Leica Microsystems, Wetzlar, Germany). Immunofluorescent or immuno histo‐morphometry quantification was performed by image J. Semi‐quantification was performed by measuring positive signals in the region of interest (ROI). Results are expressed as average optical intensity (AOD) calculated with the equation. AOD = total fluorescence intensity of ROI/area of ROI.

### 4.11 Statistical analysis

All the quantitative data were presented as mean and standard deviation. After checking of normal distribution by Kolmogorov–Smirnov test, all parameters were analyzed by ANOVA and post hoc Turkey's HSD. For histological scoring, non‐parametric Mann–Whitney *U* tests were used for comparisons between groups. The statistical analysis is calculated by SPSS (version 11; SPSS Inc, Chicago, IL, USA) and the level of significance is considered at *p* < 0.05.

## 5 CONCLUSION

In conclusion, we have developed a method to produce secretomes from fetal MSCs on a large scale and demonstrated the unique characteristics and bioactivities of HFS in vitro and in vivo. HFS‐PLGA particles may be used for promoting wound healing or tissue repair in future clinical applications. Exosomes and the heat shock protein family could be used as a functional biomarkers of the pro‐angiogenic function of the HFS.

## AUTHOR CONTRIBUTIONS

**Bin Wang:** Conceptualization (lead); data curation (lead); formal analysis (lead); funding acquisition (lead); investigation (lead); methodology (lead); project administration (lead); supervision (lead); visualization (lead); writing – original draft (lead); writing – review & editing (lead). **Mengru Pang:** Data curation (lead); formal analysis (equal); investigation (lead); methodology (lead). **Yancheng Song:** Formal analysis (supporting); funding acquisition (supporting); investigation (supporting); resources (supporting). **Haixing Wang:** Data curation (equal); formal analysis (supporting); investigation (supporting); methodology (supporting). **Pan Qi:** Data curation (supporting); formal analysis (supporting); investigation (supporting); methodology (supporting). **Shanshan Bai:** Data curation (supporting); formal analysis (supporting); funding acquisition (supporting); methodology (supporting). **Xiaoxuan Lei:** Data curation (supporting); formal analysis (supporting); funding acquisition (supporting); methodology (supporting). **ShiKun Wei:** Data curation (supporting); investigation (supporting); methodology (supporting). **Zhixian Zong:** Data curation (supporting); formal analysis (supporting). **Sien Lin:** Data curation (supporting); formal analysis (supporting). **XiaoTing Zhang:** Data curation (supporting); investigation (supporting); methodology (supporting). **XiaoTong Cen:** Formal analysis (supporting); investigation (supporting); methodology (supporting). **Xia Wang:** Data curation (supporting). **YongKang Yang:** Data curation (supporting); formal analysis (supporting); investigation (supporting); methodology (supporting). **Yuan Li:** Data curation (supporting); formal analysis (supporting); investigation (supporting); methodology (supporting). **Yan Wang:** Formal analysis (supporting); funding acquisition (supporting); methodology (supporting); resources (supporting); supervision (supporting). **Hongjie Xu:** Formal analysis (supporting); investigation (supporting); methodology (supporting); resources (supporting). **Lin Huang:** Conceptualization (supporting); formal analysis (equal); methodology (lead); resources (equal). **Micky Tortorella:** Conceptualization (supporting); funding acquisition (supporting); resources (supporting); supervision (supporting). **Biao Cheng:** Conceptualization (equal); funding acquisition (equal); resources (equal); supervision (equal). **Yukwai Lee:** Data curation (equal); funding acquisition (equal); investigation (equal); resources (equal); supervision (equal). **Dajiang Qin:** Conceptualization (equal); investigation (equal); resources (equal); supervision (equal). **Gang Li:** Conceptualization (lead); data curation (lead); project administration (lead); resources (lead); supervision (lead); writing – original draft (lead); writing – review & editing (lead).

## CONFLICT OF INTERESTS

All the authors declared no conflict of interest.

## ETHICAL STATEMENT

Human fetal MSC was isolated from first‐trimester fetal bone tissues which were stored in the Cell Bank of the Prince of Wales Hospital of the Chinese University of Hong Kong. Human ethics approval was obtained from the Joint CUHK‐NTEC Clinical Research Ethics Committee of the Chinese University of Hong Kong (Ref. No. CRE‐2011.383). Human adult MSC was isolated from adult bone tissue. The use of human adult samples was approved by the Joint CUHK‐NTEC Clinical Research Ethics Committee (Ref. No. CRE‐2010.248). For animal studies, surgery was carried out under the animal license issued by the Hong Kong SAR Government and the approval of the Animal Experimentation Ethics Committee of the Chinese University of Hong Kong (Ref No. 17‐145‐ITF).

## Supporting information


**Figure S1** Cell viability during 10‐day culture of hfMSCs in a PBS bioreactor. Cell viability was determined by cell counting. 5 million cells were expended to 30 million cells during 10 days culture.

## Data Availability

Data availability statement The data that support the findings of this study are available from the corresponding author upon reasonable request.
