# Supplementary material for: Human fetal mesenchymal stem cells secretome promotes scarless diabetic wound healing through heat‐shock protein family
Source: Bioeng Transl Med. 2022 Jun 21;8(1):e10354. doi: 10.1002/btm2.10354 (PMC9842061; doi:10.1002/btm2.10354)
Supplement: Supplementary file 1 — Figure S1 Cell viability during 10‐day culture of hfMSCs in a PBS bioreactor. Cell viability was determined by cell counting. 5 million cells were expended to 30 million cells during 10 days culture. [file BTM2-8-e10354-s001.pdf]

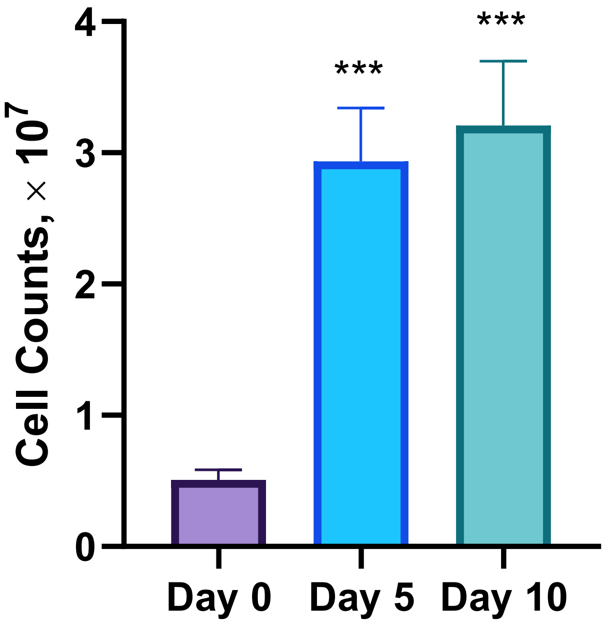

Figure S1. Cell viability during 10-day culture of hfMSCs in a PBS bioreactor. Cell viability was determined by cell counting . 5 million cells were expended to 30 million cells during 10 days culture.
